# A Label-Free Assay for Aminoacylation of tRNA

**DOI:** 10.3390/genes11101173

**Published:** 2020-10-07

**Authors:** Howard Gamper, Ya-Ming Hou

**Affiliations:** Department of Biochemistry and Molecular Biology, Thomas Jefferson University, Philadelphia, PA 19107, USA; Howard.Gamper@jefferson.edu

**Keywords:** aminoacyl-tRNA synthetases, prolyl-tRNA, tyrosyl-tRNA, biotinylation to aminoacyl-tRNA, acp^3^U47 in tRNA

## Abstract

Aminoacylation of tRNA generates an aminoacyl-tRNA (aa-tRNA) that is active for protein synthesis on the ribosome. Quantification of aminoacylation of tRNA is critical to understand the mechanism of specificity and the flux of the aa-tRNA into the protein synthesis machinery, which determines the rate of cell growth. Traditional assays for the quantification of tRNA aminoacylation involve radioactivity, either with a radioactive amino acid or with a [3′-^32^P]-labeled tRNA. We describe here a label-free assay that monitors aminoacylation by biotinylation-streptavidin (SA) conjugation to the α-amine or the α-imine of the aminoacyl group on the aa-tRNA. The conjugated aa-tRNA product is readily separated from the unreacted tRNA by a denaturing polyacrylamide gel, allowing for quantitative measurement of aminoacylation. This label-free assay is applicable to a wide range of amino acids and tRNA sequences and to both classes of aminoacylation. It is more sensitive and robust than the assay with a radioactive amino acid and has the potential to explore a wider range of tRNA than the assay with a [3′-^32^P]-labeled tRNA. This label-free assay reports kinetic parameters of aminoacylation quantitatively similar to those reported by using a radioactive amino acid, suggesting its broad applicability to research relevant to human health and disease.

## 1. Introduction

Aminoacylation of tRNA is catalyzed by aminoacyl-tRNA synthetases (aaRSs) in an ATP-dependent reaction [1]. The energy of ATP hydrolysis activates the carboxyl group of the amino acid to esterify with one of the cis-diols of the terminal A76 ribose in tRNA. The synthesized aa-tRNA embodies the genetic code, where the aminoacyl group on the 3′-end of the tRNA is physically related to the triplet anticodon on the anticodon end. This aa-tRNA readily interacts with elongation factors in the presence of GTP and the ternary complex is brought to the ribosome A site (the aa-tRNA binding site), where it is accommodated at the codon sequence complementary to the anticodon. Once accommodated, the aa-tRNA serves as the acceptor for the ribosome-catalyzed peptidyl transfer from the peptidyl-tRNA at the ribosome P site (the peptidyl-tRNA binding site). Thus, aminoacylation of tRNA is the first step in the making of a new peptide bond, which directly impacts on the overall rate of protein synthesis and hence the rates of mass accumulation and growth of cells in bacteria [2,3]. It is a reaction frequently implicated in neurological disorders and thus is highly relevant to human health and disease [4]. Additionally, some aa-tRNAs are associated with non-ribosomal cellular activities. A well-known example of the latter is post-translational arginylation to protein catalyzed by arginyl transferases, which use Arg-tRNA^Arg^ as the aminoacyl donor for transferring to protein substrates as the marker for degradation [5,6]. In both ribosome-dependent and ribosome-independent activities, the level of each aminoacylation reaction determines the flow and the amount of the aa-tRNA that supports these activities. 

Traditional assays for the aminoacylation of tRNA involve radioactivity. The most frequently used assay, which was historically the first developed (reviewed in [7]), employs a radioactive amino acid and monitors its transfer to the 3′-end of the tRNA substrate, generating a radioactive aminoacyl group on the aa-tRNA that is acid precipitable on filter pads and can be quantified in a scintillation counter. The major drawback of this assay is that, because the label is on the amino acid, the assay is performed with limiting concentrations of the amino acid (10–20 µM) to provide the sensitivity necessary for detection, which cannot satisfy the *K*_m_ of the amino acid for tRNA aminoacylation (200–400 µM) [8,9,10,11,12,13]. This constraint prevents elucidation of kinetic parameters of the tRNA in saturating concentrations of the amino acid. In a more recently developed aminoacylation assay [14], the tRNA is ^32^P-labeled at the 3’-end, using a CCANo further definition is needed.-adding enzyme that catalyzes the removal of the terminal A76 nucleotide and addition of [α-^32^P]-ATP to repair the 3′-end [15]. Following aminoacylation, the [3′-^32^P]-labeled aa-tRNA is digested to mononucleotides, such that the labeled terminal AMP carrying the aminoacyl group (aa-[^32^P]AMP) can be separated from the labeled terminal AMP lacking the aminoacyl group ([^32^P]AMP) by thin layer chromatography (TLC). The fraction of aa-[^32^P]AMP is then directly monitored and quantified. While this assay is performed with saturating concentrations of the amino acid, thus overcoming the deficiency of the assay with a labeled amino acid, it remains insufficient to assess variant tRNAs that have poor binding affinity to the aaRS of interest. The difficulty with poorly binding tRNAs lies in the need to maintain a sufficiently low tRNA concentration to retain the sensitivity of detection. While the low concentration satisfies the *K*_m_ of tRNAs of wild-type sequences (0.2–2.0 µM) [11,13,16,17,18,19,20], it cannot satisfy the *K*_m_ of variant tRNAs that have lost the binding affinity by more than 10-fold, including those harboring pathogenic mutations and those that have evolved across biological domains [17,18,21]. This limitation on tRNA concentration prevents the investigation of how pathogenic mutations in tRNA affect aminoacylation, or how evolution has altered the dynamics between an aaRS and its tRNA substrate. Moreover, with both radio-labeling assays, whether placing the label on the amino acid or tRNA, the costs and extra procedures of handling radioactive reagents are additional factors of inconvenience. 

Although two new assays for the aminoacylation of tRNA were developed recently, these are designed to analyze a specific tRNA relative to all tRNAs in a population. In the microarray assay [20,22,23,24], the unreacted fraction of a specific tRNA is removed by periodate oxidation, and the aa-tRNA fraction is deacylated, joined at the 3’-end with a fluorophore-labeled oligonucleotide, and hybridized to a microarray that contains a probe complementary to the tRNA. The level of aa-tRNA fraction is not quantified relative to its unreacted fraction but is quantified relative to all other aa-tRNAs on the microarray. In the deep-sequencing assay [25,26], the unreacted fraction of a specific tRNA is removed by periodate oxidation, and the aa-tRNA fraction is quantified by deep-sequencing and compared in levels relative to those of other aa-tRNAs in the population. Given that these two recent assays cannot determine the aminoacylation of a specific tRNA relative to its unreacted fraction, and that the two radio-labeling assays are limited with respect to substrate concentration, we describe here a label-free assay that overcomes the shortcomings of the available assays. This label-free assay is based on the principle developed in a previous assay, in which the α-amine or the α-imine of the aminoacyl group of an aa-tRNA is biotinylated and then conjugated with streptavidin (SA), forming a biotin-SA-conjugate that is readily separated from the uncharged tRNA in a denaturing PAGE/7 M urea gel [27,28]. We demonstrate that this label-free assay is applicable to a wide range of substrates, including amino acid analogs that are non-proteinogenic and tRNA species that are synthesized in cells and are present within the entire pool of all other cellular tRNAs. A distinct feature of tRNAs synthesized in cells (termed as in the native state) is the decoration by cellular enzymes with post-transcriptional modifications that substantially expand the chemical diversity of nucleotides. With one such tRNA as an example, we report that the label-free assay has the sensitivity and accuracy needed to determine the kinetic parameters of aminoacylation at levels similar to those reported for the tRNA in the purified state.

## 2. Materials and Methods

### 2.1. Isolation of tRNAs 

All tRNAs used in this study were of the *Escherichia coli* origin and most were native tRNAs that had been deacylated in pH 9.0 buffer prior to use. The identity and anticodon sequence, located at nucleotide positions 34–36, of each is tRNA^fMet^ (the initiator tRNA, anticodon CAU), tRNA^Arg^ (anticodon ACG, where the wobble A34 is modified to inosine (I) in the native state), tRNA^Tyr^ (anticodon GUA, where the wobble G34 is modified to queuosine34 (Q34) in the native state), tRNA^Val^ (anticodon UAC, where the wobble U34 is modified to cmo^5^U34 in the native state), tRNA^Ala^ (anticodon GGC), tRNA^Lys^ (anticodon UUU, where the wobble U34 is modified to mnm^5^s^2^U34 in the native state), tRNA^Pro^ (anticodon GGG), and tRNA^Ser^ (anticodon GCU). All of these tRNAs in the native state were expressed from an inducible and overexpression clone made in the pKK223-3 plasmid, respectively [17]. For *Escherichia coli* tRNA^fMet/CAU^, tRNA^Arg/ICG^, tRNA^Tyr/QUA^, tRNA^Val/cmo5UAC^, and tRNA^Lys/mnm5s2UUU^, respectively, the level of overexpression of each in the native state was high, accounting for over 30% of the total tRNA, such that each was isolated together with the pool of *E. coli* tRNAs as an overrepresented species. For *E. coli* tRNA^Ala^ and tRNA^Pro^, respectively, the level of overexpression of the native state was low, accounting for <10% of the total tRNA, requiring each to be purified by hybridization to a biotin-tagged oligonucleotide that was attached to an SA-conjugated solid support, followed by elution from the solid support with two consecutive steps of heat treatment at 65 °C for 5 min each [18]. 

### 2.2. Isolation of E. coli aaRS Enzymes

Recombinant His-tagged *E. coli* class I enzymes MetRS, ArgRS, TyrRS, and ValRS, and class II enzymes AlaRS, LysRS, ProRS, and SerRS, were each purified from an overexpression clone based on the pET plasmid system in *E. coli* BL21 (DE3) [29]. All proteins were determined for concentration by the Bradford assay and were stored in 50 mM Tris-HCl, pH 7.5, 1 mM dithiothreitol (DTT), and 40% glycerol at −20 °C. 

### 2.3. Saturation Level of Aminoacylation of tRNA 

Aminoacylation of tRNA was performed using the cognate aaRS and amino acid in 30 µL of 20 mM Tris-HCl pH 7.5 buffer containing 20 mM KCl, 10 mM MgCl_2_, 10 mM DTT, and 0.1 µg/µL BSA as described [29,30]. The concentration of each input tRNA was determined by absorption at A260 on a NanoDrop instrument. Reactions were incubated at 37 °C for 12 min with each tRNA sample (10 µM, heat-cooled in 10 mM Tris-HCl, pH 7.5, and 10 mM MgCl_2_), 20 µM aaRS, 350 µM amino acid, and 6 mM ATP. The product aa-tRNA of each reaction was extracted with pH 5.0 phenol, ethanol precipitated, and the pellet dissolved in 30 µL H_2_O. Equal 15 µL portions were immediately analyzed for saturation level of aminoacylation by measuring the labeled aminoacyl moiety on the aa-tRNA or by conjugation of the aminoacyl moiety with biotin-SA as previously reported [27,28]. For quantification of the saturation level of aminoacylation using a radioactive amino acid, a trace amount of the labeled amino acid was spiked into the aminoacylation reaction as above, and an aliquot of 1 µL of each reaction was removed and added into 99 µL of water, from which 5 µL was counted in a scintillation counter to determine the specific activity of the amino acid. The specific activity determined as such was 23,580 dpm/pmole for Met, 1273 dpm/pmole for Arg, 1556 dpm/pmole for Tyr, 392 dpm/pmole for Val, 1232 dpm/pmole for Ala, 1419 dpm/pmole for Lys, and 1468 dpm/pmole for Pro. After aminoacylation, a 15 µL aliquot of each reaction was diluted 2-fold with 50 mM sodium acetate (NaOAc), pH 5.0, and the 30 µL was passed through a CentriSpin 20 cartridge (Princeton Separation, Adelphia, NJ, USA) hydrated in 25 mM NaOAc, pH 5.0. To the flow-through, which contained the aa-tRNA, the absorption at A_260_ was measured in a NanoDrop to determine the concentration of total tRNA. The radioactivity was determined by counting 5 µL in a scintillation counter and was converted to pmoles for the aa-tRNA based on the specific activity of the amino acid. The saturation level of tRNA aminoacylation was calculated from the amount of aa-tRNA relative to the amount of total tRNA in the flow-through. 

### 2.4. Flexizyme-Catalyzed Aminoacylation of E. coli tRNA^Ser^ with Non-proteinogenic Amino Acids 

Reactions were performed as described [27,28]. Briefly, a reaction (11 µL final volume) containing 30 µM tRNA^Ser^ transcript (made by in vitro transcription with natural NTPs), 30 µM dFx flexizyme [27], 3 mM aminoacyl-DBE (where DBE = 3,5-dinitrobenzyl ester), and 330 mM MgCl_2_ in 60 mM HEPES pH 7.5 with 12.5% DMSO was incubated in an ice bath for 30 min (for *cis*-hydroxyproline-DBE, *trans*-hydroxyproline-DBE, and azetidine-DBE), for 2 h (for citrulline-DBE), or for 4 h (for serine-DBE and NEM-cysteine-DBE). Reactions were quenched with 100 µL of 250 mM NaOAc pH 5.0, ethanol precipitated, and each pellet dissolved in 34 µL H_2_O. An aliquot of 15 µL was immediately biotinylated as described below.

### 2.5. Biotin-SA Conjugation 

Biotinylation of the α-amine or α-imine of an aa-tRNA was modified from a previous assay [27,28] and was carried out at 4 °C, which was found to more quantitatively capture the aa-tRNA product than at room temperature. Reactions utilized 15 µL of each aa-tRNA and were performed for 1 h in 60 µL of 60 mM HEPES, pH 8.0, with approximately 2.5 µM of tRNA and 15 mM *N*-hydroxy-sulfo-succinimido-biotin (sulfo-NHS-biotin, Thermo Fisher, Waltham, MA, USA). Following two consecutive ethanol precipitations, each with a 70% ethanol wash, the biotinylated aa-tRNA pellet was dissolved in 12 µL water. A 1.0 µL aliquot of the biotinylated aa-tRNA (~8 pmoles) was incubated with 4 µL of 1 mg/mL SA (72 pmoles) for 20 min at room temperature and loaded onto an analytical denaturing 12% PAGE/7M urea gel run at 200 V for 50 min. The 9-fold molar excess of SA over aa-tRNA led to the formation of predominantly one SA conjugated to one aa-tRNA, resulting in one major biotin-SA-conjugated aa-tRNA complex. Bands of the biotin-SA-tRNA and the unreacted tRNA were visualized by staining with SYBR gold, and the fraction of aa-tRNA in the input tRNA was calculated. 

### 2.6. Determination of Kinetic Parameters. 

The active concentration of the overrepresented *E. coli* tRNA^Tyr^ in the entire pool of *E. coli* tRNAs was used in a series of kinetic assays, ranging from 0, 0.4, 0.8, 1.6, 3.2, to 6.4 µM at the constant *E. coli* TyrRS concentration of 1 nM over a time course of 2, 4, 6, and 8 min for each concentration. Prior to each reaction, *E. coli* tRNA^Tyr^ was heated at 85 °C for 2 min, annealed at 37 °C for 15 min in a heat-cool solution (10 mM Tris-HCl, pH 7.5, 10 mM MgCl_2_) and was added to a reaction of final volume of 24 µL of 20 mM KCl, 50 mM Tris-HCl, pH 7.5, 4 mM DTT, 0.15 mg/mL BSA, 10 mM MgCl_2_, 2 mM ATP, 0.2 mM Tyr at 37 °C. Purified *E. coli* TyrRS (6 nM) was added in 4 µL to bring the total volume to 24 µL. At specified time points, an aliquot of 4 µL from each reaction was removed and mixed with 6 µL of a quench solution to the final volume of 10 µL in 0.25 M NaOAc, pH 5.0, glycogen at 0.08 µg/µL, and 10 mM EDTA. The quenched solution was ethanol precipitated, dissolved in water, and an aliquot containing 4.5 pmoles tRNA (of which 35% was tRNA^Tyr^) was processed by biotinylation, SA conjugation, and denaturing gel analysis in 12% PAGE/7 M urea. Data were fit to the Michaelis–Menten equation to derive the parameters *K*_m_ (tRNA), *k*_cat_, and *k*_cat_/*K*_m_ (tRNA).

## 3. Results 

### 3.1. A Label-Free Assay for Aminoacylation of tRNA by Biotin-SA Conjugation

**Figure 1:**
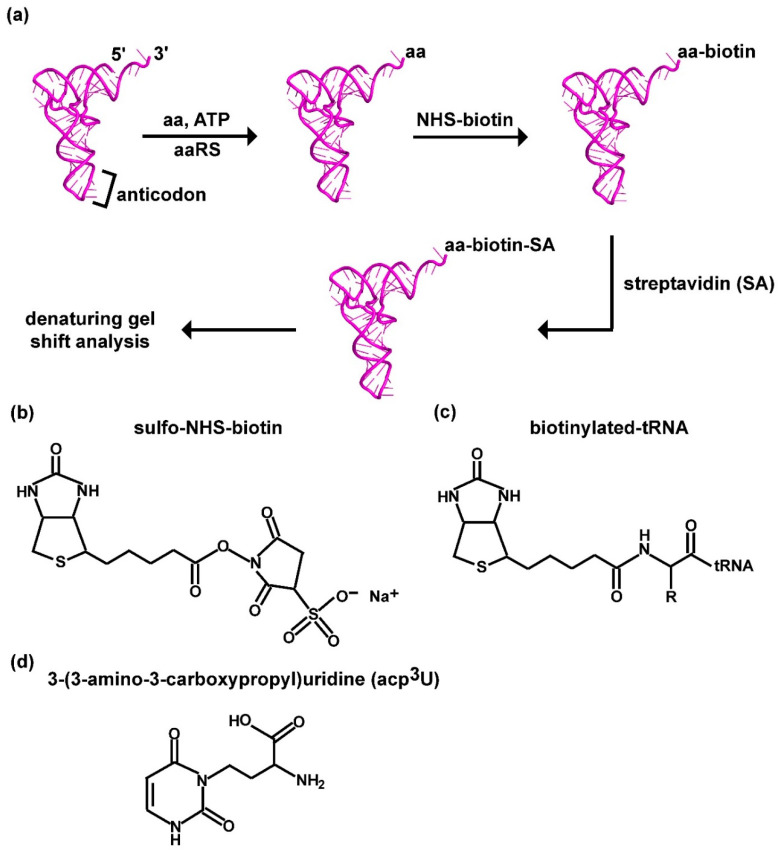
A label-free assay for aminoacylation of tRNA based on biotin-SA conjugation to the aminoacyl group. (**a**) A scheme of the label-free assay, starting with aminoacylation of tRNA catalyzed by an aaRS in the presence of an amino acid and ATP, followed by biotinylation of the aminoacyl group in the aa-tRNA, SA conjugation to biotin, and denaturing PAGE/7M urea gel to separate the product from the unreacted tRNA. (**b**) Chemical structure of sulfo-NHS-biotin. (**c**) Chemical structure of biotinylation to the aminoacyl group of an aa-tRNA, where R is the side chain of the amino acid. (**d**) Chemical structure of acp^3^U in tRNA, showing the presence of a primary amine that would be reactive to biotin. Abbreviations: aa: amino acid; SA: streptavidin; acp^3^U: 3-(3-amino-3-carboxypropyl) uridine.

The principle of the label-free assay (Figure 1a) is derived from a previous report that aminoacylation of tRNA generates an alkyl α-amine associated with the carbonyl moiety of the aminoacyl group at the 3′-end of the aa-tRNA, which is reactive in nucleophilic attack on the ester of the NHS ring of sulfo-NHS-biotin (Figure 1b), forming an amide linkage (Figure 1c) while releasing the NHS group [27,28]. The amide bond is stable and cannot be cleaved and, through its linkage to biotin, can be quantified by an SA-binding assay. Notably, although the aminoacyl linkage is highly labile and is rapidly hydrolyzed in neutral pH or higher, biotinylation to the alkyl-amine stabilizes this linkage and permits binding with SA to generate a final product of biotin-SA-conjugated aa-tRNA that would migrate substantially slower relative to the unreacted tRNA in a typical denaturing PAGE/7 M urea gel at pH 8.3. Without biotinylation, the separation of an aa-tRNA from the unreacted tRNA is more difficult, requiring radio-labeling of the tRNA and gel electrophoresis in an acid buffer at pH 5.0 [30,31]. 

To successfully use this biotin-SA conjugation assay to monitor the aminoacylation of tRNA, however, two key points need to be considered. First, due to the diverse chemical structures of proteinogenic amino acids, whether the biotin-SA conjugation method is broadly applicable to these structures is unknown. Additionally, aminoacylation of tRNA takes place in two distinct mechanisms [29,32], where class I aminoacylation trans-esterifies the aminoacyl moiety to the 2′-hydroxyl of the terminal ribose in tRNA, while class II aminoacylation trans-esterifies to the 3′-hydroxyl [29,32]. While the generated aminoacyl group rapidly migrates between the two diols in both directions without enzyme catalysis, the 3′-aminoacyl group is stabilized by interaction with the translation elongation factor EF-Tu in complex with GTP in bacteria (eEF2 in eukaryotes) [29,33,34]. In the absence of EF-Tu-GTP, how the dynamics and equilibrium of the migration and whether one position is more favorable for biotin-SA conjugation are unknown. Second, for biotin-SA conjugation to specifically quantify the aminoacyl group in aa-tRNA, it cannot react with tRNA nucleotides. While tRNA nucleobases also contain primary amines (e.g., the 4-amine of adenosine, the 2-amine of guanosine, and the 4-amine of cytosine), these amines are associated with aromatic rings, rendering their electrons delocalized and much less nucleophilic toward the reactive NHS-ester. However, while tRNA nucleobases indeed do not react with NHS-ester [27], some natural post-transcriptional modifications to tRNA synthesized in cells contain a primary amine that is reactive with NHS-ester [35]. A notable example is the 3-(3-amino-3-carboxy propyl) (acp^3^) modification to uridine [36,37] (Figure 1d), which contains an alkyl-amine on the carboxy propyl side chain of the modified uridine that serves as a reactive site with Cy3- or Cy5-NHS for fluorescence labeling of tRNA [38]. Whether this acp^3^-modified uridine, which usually occurs in the highly compact tertiary core structure of tRNA at position 47 in the V-loop or position 20 in the D-loop [39], is as accessible to the biotin-SA conjugation as the alkyl-amine of the aa-tRNA is unknown. Below, we address both key points in our development of the biotin-SA conjugation assay, using *E. coli* tRNAs and *E. coli* aaRS enzymes as a model. 

### 3.2. The Label-Free Assay Is Applicable to a Wide Range of Amino Acids and tRNAs

**Figure 2:**
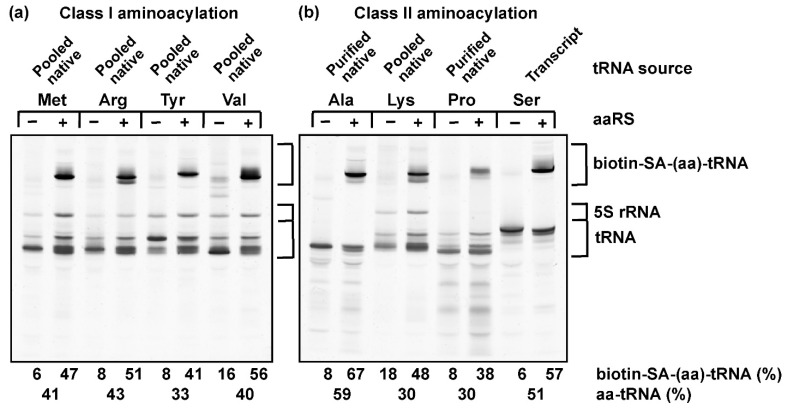
Separation of biotin-SA-conjugated aa-tRNA from unreacted tRNA. (**a**) Products of aa-tRNAs generated by class I aminoacylation with Met to tRNA^fMet/CAU^, Arg to tRNA^Arg/ICG^, Tyr to tRNA^Tyr/QUA^, and Val to tRNA^Val/cmo5UAC^. (**b**) Products of aa-tRNAs generated by class II aminoacylation with Ala to tRNA^Ala/GGC^, Lys to tRNA^Lys/mnm5s2UUU^, Pro to tRNA^Pro/GGG^, and Ser to tRNA^Ser/GCU^. All tRNAs were of the *E. coli* origin. “Pooled native” denotes that the tRNA was overexpressed in *E. coli* and thus in the native state and was isolated with the entire pool of *E. coli* tRNAs, in which it was an overrepresented species. The % of aminoacylation as quantified by the biotin-SA capturing method revealed the level of its representation in the entire pool. “Purified native” denotes that the tRNA was overexpressed in *E. coli*, and thus in the native state, isolated with the entire pool of *E. coli* tRNAs, and purified from the pool by binding to, and elution from, a biotin-tagged complementary oligonucleotide bound to an SA-conjugated solid support. The % of aminoacylation as quantified by the biotin-SA capturing method revealed the level of its representation in the purified fraction. “Transcript” denotes that the tRNA was synthesized by in vitro transcription with T7 RNA polymerase using all four natural NTPs as the substrates and was in the transcript state. The % of aminoacylation in each reaction was calculated by quantifying all of the upshifted species as the sum over the input tRNA and by subtracting out the % in the background observed in the control lane run in parallel but lacking aminoacylation.

To test the range of applicability of the biotin-SA conjugation in the label-free assay, we selected four amino acids, Met, Arg, Tyr, and Val, for aminoacylation by class I enzymes (Figure 2a), of which Met was used to aminoacylate the initiator tRNA^fMet^. We also selected four amino acids, Ala, Lys, Pro, and Ser, for aminoacylation by class II enzymes (Figure 2b). These eight amino acids differ from each other in having an aliphatic, aromatic, sulfur-containing, or an ε-amine-containing side chain. The cognate tRNA of each amino acid was prepared in the transcript form (for tRNA^Ser/GCU^, where GCU is the anticodon), in the native form purified from the total pool of *E. coli* tRNAs (for tRNA^Ala/GGC^ and tRNA^Pro/GGG^), or in the overrepresented native form in the presence of the total pool of *E. coli* tRNAs (for tRNA^fMet/CAU^, tRNA^Arg/ICG^, tRNA^Tyr/QUA^, tRNA^Val/cmo5UAC^, and tRNA^Lys/mnm5s2UUU^). These eight tRNAs provided a broad spectrum of nucleotide sequences and post-transcriptional modifications. For example, the analysis of tRNA^Arg/ICG^ and tRNA^Lys/mnm5s2UUU^, which are the only two of the eight that contain an acp^3^U47 [39], would provide insight into the accessibility of this post-transcriptional modification to biotin-SA conjugation.

Aminoacylation of each tRNA with the cognate amino acid in saturating concentrations was performed by the cognate aaRS to completion. The synthesized aa-tRNA was biotinylated, SA-conjugated, and run on a denaturing 12% PAGE/7M urea gel. To determine the reactivity of post-transcriptional modifications in each tRNA to biotin-SA conjugation, a separate reaction in the absence of the enzyme was performed in parallel. Although SA is a tetramer and capable of binding to multiple biotinylated aa-tRNA molecules to generate a heterogeneous population of biotin-SA-conjugated aa-tRNAs, we primarily observed a major upshifted band, due to the molar excess of SA to aa-tRNA (9:1, Materials and Methods) in our reaction condition. Nonetheless, all of the upshifted bands were added up to represent the collective fraction of total tRNA that was accessible to biotin-SA conjugation. Subtraction of the fraction observed in the control without aminoacylation from the fraction obtained after aminoacylation revealed the reactivity of the alkyl-amine of the aminoacyl group to the biotin-SA conjugation.

The results showed a low background of reactivity to biotin-SA conjugation in control reactions, ranging from 6% for tRNA^Ser/GCU^ and tRNA^fMet/CAU^ to 16% for tRNA^Val/cmo5UAC^ and 18% for tRNA^Lys/mnm5s2UUU^. Between tRNA^Arg/ICG^ and tRNA^Lys/mnm5s2UUU^, which contained an acp^3^U47, the background of tRNA^Lys/mnm5s2UUU^ at 18% was more noticeable than that of tRNA^Arg/ICG^ at 8%. Thus, despite being placed at the same position, the chemical reactivity of acp^3^U47 to biotin-SA conjugation can differ depending on the tRNA sequence. Although both tRNA^Arg/ICG^ and tRNA^Lys/mnm5s2UUU^ were overexpressed in cells, the level of post-transcriptional modification to synthesize acp^3^U47 was likely stoichiometric under the expression condition, as evidenced by mass spectrometry quantification of other post-transcriptional modifications [40]. In contrast to the low reactivity of acp^3^U47, the reactivity of the alkyl-amine of the aminoacyl group after aminoacylation was robust. By quantifying the level of biotin-SA conjugation, we showed that the aminoacylation efficiency ranged from 30% for tRNA^Pro/GGG^ to 59% for tRNA^Ala/GGC^ after subtraction of background. Thus, regardless of the chemical structure of the amino acid and the class I or class II mechanism of aminoacylation, the aa-tRNA product of each tRNA was much more reactive to biotin-SA conjugation than any post-transcriptionally modified tRNA nucleotides (including acp^3^U47). Of interest was successful conjugation of biotin-SA to the product of aminoacylation of tRNA^Pro/GGG^, which contains an α-imine, indicating that even this secondary amine is accessible, consistent with a previous report [41]. Thus, the biotin-SA capturing of aa-tRNA is broadly applicable to a wide range of amino acids and tRNA sequences, including those in the latter with diverse post-transcriptionally modified nucleotides. 

### 3.3. Stoichiometry of Quantifying Aminoacylation by the Label-Free Assay

We next tested whether the label-free assay captured the stoichiometry of aminoacylation and whether its quantification was comparable to that of using a radioactive amino acid. For each aminoacylation reaction, we summed up all of the upshifted bands and calculated the total fraction that was reactive to biotin-SA conjugation and subtracted it from the background fraction observed in the control reaction without aminoacylation (Table 1). As an independent measurement of aminoacylation, each tRNA was also assayed by the traditional method with the radioactive form of the amino acid. We found that while the aminoacylation reaction was performed at 37 °C, this temperature was not optimal for the biotinylation reaction, likely due to competition between hydrolysis of the aminoacyl linkage and biotinylation of the amino acid. Competition was suppressed when biotinylation was performed at 4 °C [27], where the level of aminoacylation quantified by biotin-SA conjugation was at least equal to, or more likely higher by almost two-fold than, the level quantified with a labeled amino acid (Table 1). This observation was consistent across all cases, indicating that the biotin-SA conjugation method is quantitatively and stoichiometrically more sensitive than the assay with a labeled amino acid, capturing more of the product of aminoacylation. It also points to the general deficiency of quantification with labeled amino acids, due to the inherent difficulty of precise determination of radioactivity. Notably, the calculated yield of aminoacylation for tRNAs that existed as an overrepresentative species in the pool of *E. coli* tRNAs (i.e., Met-tRNA^fMet/CAU^, Arg-tRNA^Arg/ICG^, Tyr-tRNA^Tyr/QUA^, Val-tRNA^Val/cmo5UAC^, and Lys-tRNA^Lys/mnm5s2UUU^) included the small fraction of other isoacceptors that were expressed at the native level (~2% of the total pool of *E. coli* tRNAs) and were aminoacylated by the respective cognate aaRS enzymes. This small overrepresentation of aminoacylation applied to both the biotin-SA conjugation assay and the assay with a labeled amino acid.

### 3.4. The Label-Free Assay Is Applicable to Non-Proteinogenic Amino Acids

We tested the ability of the biotin-SA conjugation method to assess aminoacylation with non-proteinogenic amino acids, which are usually not available in a radioactive form and thus cannot be assayed by the traditional assay. While custom synthesis of each non-proteinogenic amino acid in the radioactive form would be extremely costly, the impact of investigating the aminoacylation of each is high in synthetic chemistry, particularly with the goal to “recode” the genome, which is currently limited to the 20 proteinogenic amino acids that compose cellular proteins. The ability to recode the genome and to expand the chemical repertoire of proteins to include non-proteinogenic amino acids promises to offer novel tools to probe protein structure and function [42]. Several aaRS enzymes have been engineered to catalyze the aminoacylation of tRNA with non-proteinogenic amino acids, thus generating products that can be delivered to a site-specific position during protein synthesis [42]. In the absence of a radioactive non-proteinogenic amino acid, measurement of aminoacylation would require a labeled tRNA [14]. To test the feasibility of the label-free assay for aminoacylation of tRNA with non-proteinogenic amino acids, we selected several analogs of amino acids that lack a cognate aaRS as a general approach. Because only a limited number of non-proteinogenic amino acids can be charged to tRNA by an aaRS protein enzyme [42], the general strategy by which to perform aminoacylation is to use the RNA-based dFx flexizyme [27], which recognizes the universal CCA sequence of tRNA at the 3’-end by base-pairing interaction and catalyzes transfer of the activated form of each amino acid as a dinitro-benzyl ester (DBE) [43,44]. Due to the lack of discrimination of dFx flexizyme among tRNA primary sequences [44], the tRNA substrate for quantification of aminoacylation should be homogeneous. Any contaminating tRNAs would also be aminoacylated by the flexizyme. 

**Figure 3:**
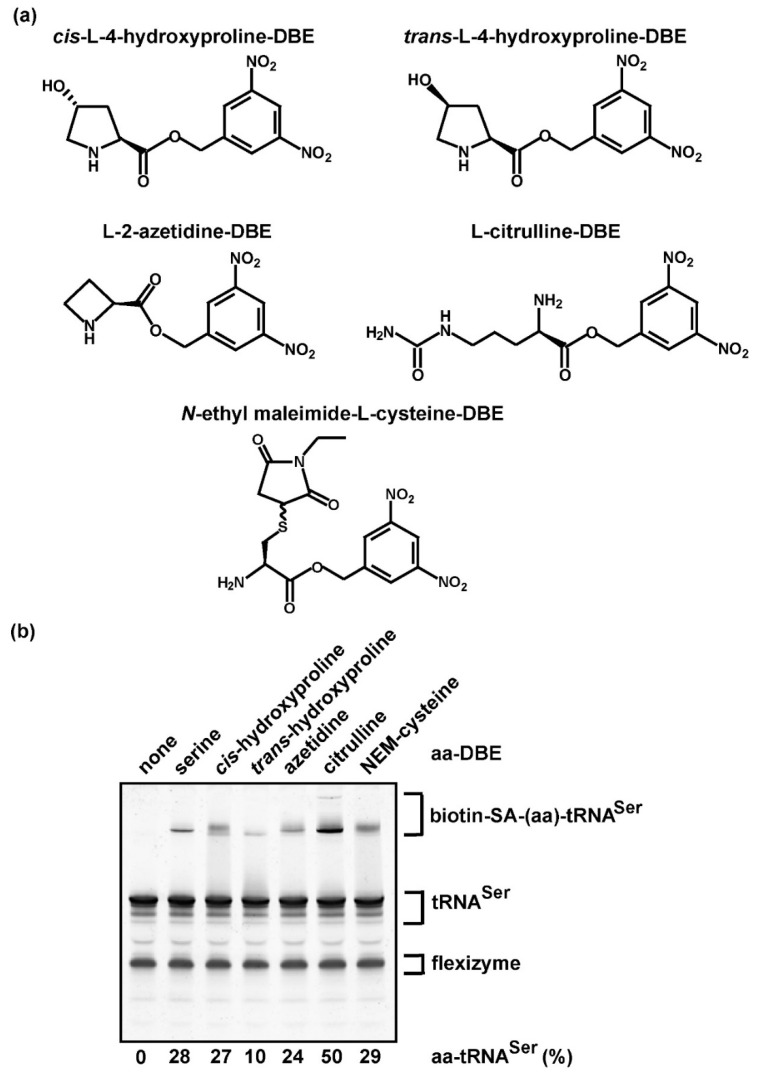
Aminoacylation with non-proteinogenic amino acids as quantified by biotin-SA conjugation. (**a**) Chemical structure of each non-proteinogenic aminoacyl-DBE used for the dFx flexizyme-catalyzed aminoacylation to the transcript of *E. coli* tRNA^Ser/GCU^. (**b**) Gel separation and quantification of the aa-tRNA product by biotin-SA conjugation. The % of aminoacylation in each reaction was calculated by adding up all of the upshifted bands relative to the input tRNA and by subtracting the % in the background observed in the control reaction run in parallel but lacking aminoacylation (in lane 1).

We chose *E. coli* tRNA^Ser/GCU^ from the collection above as the substrate (Figure 2b), which was readily available in large quantities in the purified transcript form. As a proof of principle, we selected the following amino acid analogs (Figure 3a): *cis*-hydroxyproline, *trans*-hydroxyproline, azetidine-2-carboxylic acid (abbreviated as azetidine, an analog of proline), citrulline (a precursor of arginine), and N-ethylmaleimide (NEM)-cysteine (where the NEM group is attached to the sulfur of cysteine), each of which was previously chemically synthesized as a DBE-conjugate and was readily trans-esterified to tRNA by dFx flexizyme [6,45]. Aminoacylation of *E. coli* tRNA^Ser/GCU^ with each analog, followed by biotin-SA conjugation and analysis by a denaturing/7 M urea gel, showed that all of the non-proteinogenic aa-tRNA products were captured by biotin-SA conjugation, resulting in upshifted bands, whereas the control reaction lacking an aa-DBE had no shift (Figure 3b). While the yield of aminoacylation with Ser as a control was 28%, lower than that of 51% reported above (Figure 2b), this was due to the use of dFx flexizyme for aminoacylation rather than the SerRS enzyme. For non-proteinogenic amino acids (Table 1), the yields quantified by biotin-SA conjugation were similar to those reported previously with labeled tRNA [45]. Thus, aminoacylation of tRNA with non-proteinogenic amino acids, catalyzed by dFx flexizyme, can be detected and quantified by biotin-SA conjugation, without relying on custom synthesis of the radioactive form of each. 

### 3.5. The Label-Free Assay Determines Kinetic Parameters of Aminoacylation

We next determined whether the label-free assay was able to generate kinetic parameters of aminoacylation, which would shed light on the mechanism of aminoacylation and would be particularly important to elucidate how the mechanism is perturbed by mutations. The most commonly sought after kinetic parameters of aminoacylation are the *K*_m_ of the tRNA, the concentration that produces the half-maximum rate, the *k*_cat_, the catalytic turnover of the enzyme in a multi-turnover steady-state reaction, and the derived *k*_cat_/*K*_m_ (tRNA), the catalytic efficiency of aminoacylation. These are inherent parameters of an aminoacylation reaction that can be compared between experiments and between labs. We used the overrepresented *E. coli* tRNATyr in the presence of the entire pool of *E. coli* tRNAs (Figure 2a) to test the feasibility of the label-free assay for elucidation of these parameters. The presence of the pool of *E. coli* tRNAs provided a framework within which to test the specificity of the assay. 

**Figure 4:**
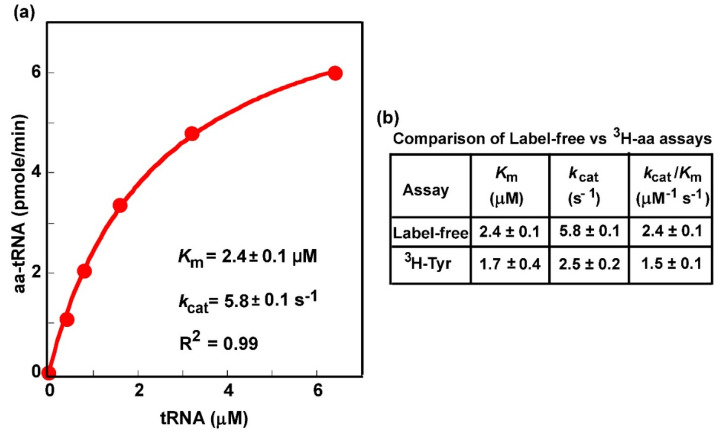
Michaelis–Menten kinetics of aminoacylation as monitored by the label-free biotin-SA conjugation assay. (**a**) A plot of the initial rate of aminoacylation as a function of the concentration of the tRNA substrate, which was the overrepresented *E. coli* tRNA^Tyr/QUA^ in the presence of the entire pool of *E. coli* tRNAs. Data were fit to the Michaelis–Menten equation to generate the hyperbolic plot to derive the value of *K*_m_ (tRNA) and *k*_cat_, showing an *R*^2^ of 0.99. (**b**) A comparison of kinetic parameters between the label-free assay as derived in (**a**) and the traditional assay with [^3^H]-Tyr as reported previously [46].

The active concentration of *E. coli* tRNA^Tyr^ for aminoacylation by *E. coli* TyrRS was varied in a series of reactions in the presence of saturating Tyr. The synthesized Tyr-tRNA^Tyr^ was quenched in acid at specific time points, subjected to biotinylation and SA conjugation, and was separated from the unreacted tRNA^Tyr^ and all other tRNA species by a denaturing gel (Figure S1). Fitting the data of synthesis of Tyr-tRNA^Tyr^ as a function of the concentration of tRNA^Tyr^ to the Michaelis–Menten equation revealed the signature of saturation kinetics (Figure 4a), with a well-fit linear regression coefficient of determination *R*^2^ = 0.99, indicating that the reaction reached the maximal velocity when all of the TyrRS molecules were in the enzyme-tRNA complex. This concentration-dependent titration produced a *K*_m_ (tRNA) of 2.4 ± 0.1 µM, a *k*_cat_ of 5.8 ± 0.1 s^−1^, and the *k*_cat_/*K*_m_ (tRNA) of 2.4 ± 0.1 M^−1^s^−1^, which were closely similar to the values previously reported for purified *E. coli* tRNATyr in the native state using the assay with [^3^H]-Tyr [46] (Figure 4b). These data demonstrate the ability of the label-free assay to specifically monitor aminoacylation of tRNA^Tyr^ in the presence of the pool of *E. coli* tRNAs and to generate kinetic parameters quantitatively similar to those generated by the traditional assay on purified tRNA^Tyr^ with [^3^H]-Tyr.

## 4. Discussion

Here, we present a label-free assay that directly quantifies the aminoacylation of tRNA by monitoring the aminoacyl group of the product with biotin-SA conjugation. We report that this label-free assay is applicable to a wide range of amino acids and tRNA sequences and that it is even applicable to single tRNA species that are present in the entire pool of cellular tRNAs. Without relying on radioactive labeling, this assay is cost-effective and circumvents many of the issues faced with using radioactive amino acids or tRNAs. It is readily accessible to non-proteinogenic amino acids and to tRNA species that are fully post-transcriptionally modified or lack any modification. It has the sensitivity and accuracy necessary for quantitative measurement of kinetic parameters of aminoacylation.

The label-free assay is designed for in vitro analysis of aminoacylation of a tRNA, either purified or partially purified from cell lysates lacking other α-amine- or α-imine-containing molecules, such as free amino acids. This limitation is similarly applicable to the assay with a labeled amino acid. To determine the level of aminoacylation of a specific tRNA in cells in an in vivo condition, the only assay is the use of acid gel, which separates aa-tRNA from unreacted tRNA by migration [30,31], followed by probing the specific tRNA in a Northern blot analysis, using a ^32^P-labeled oligonucleotide probe specific for the tRNA [47]. Thus, to quantify the level of aminoacylation in vivo, a labeling reaction is necessary among the current assays. Nonetheless, it is possible to develop a label-free assay for this purpose in the future. This would require affinity isolation of the tRNA in acid condition, followed by the biotin-SA conjugation assay to quantify the level of the aa-tRNA fraction. 

The key consideration of the label-free assay is the presence of additional primary amines, outside of the alkyl-amine of the aa-tRNA product, that may react with biotin-SA conjugation. Among amino acids, Lys contains an ε-amine that is reactive and is the basis for a common method of protein labeling [48], whereas Arg has a guanidinium group that is usually non-reactive. Neither of these alkyl-amines in the two amino acids interferes with the label-free assay in the conditions described here. Among tRNA nucleotides, while natural nucleotides are not substrates for biotin-SA conjugation, the post-transcriptionally modified acp^3^U does contain a primary amine that is weakly reactive. In *E. coli*, acp^3^U is present at position 47 in the V-loop of tRNA^Arg/ICG^ (Arg2), tRNA^Ile/GAU^ (Ile1 and Ile2), and tRNA^Ile/K2C^ (Ile2v, where K is lysidine attached to the 2-carbonyl of C), tRNA^Lys/mnm5s2UUU^, tRNA^Met/CAU^ (the elongator), tRNA^Phe/GAA^, and tRNA^Val/GAC^ (Val2A and Val2B), whereas in eukaryotes, it is at position 20 and 20a in the D-loop of several cytoplasmic tRNAs [39]. Possibly due to the highly compact local structure of the tRNA V-loop, which prevents chemical accessibility, the primary amine of acp^3^U47 in *E. coli* tRNA^Arg/ICG^ and tRNA^Lys/mnm5s2UUU^ does not readily conjugate to biotin-SA (Figure 2a,b). Nonetheless, because of the potential reactivity with post-transcriptional modifications in tRNAs, the calculation of the aa-tRNA fraction should include all of the upshifted bands and should subtract the fraction in the background observed in a parallel reaction lacking aminoacylation. These data correction steps are designed to not only improve the accuracy of the assay but also to provide insight into the chemical reactivity of tRNA post-transcriptional modifications to biotin-SA conjugation. 

Another consideration is that, while the aminoacyl group of aa-tRNA is unstable and readily hydrolyzed at neutral pH or above, the biotinylation reaction is most efficiently achieved at pH 8.0. To develop an optimal balance between the two opposing factors, we find that performing biotinylation at 4 °C as previously reported [27] can significantly improve the yield relative to reaction at room temperature for both proteinogenic and non-proteinogenic amino acids (Table 1). In all cases, we show a noticeable improvement in the level of biotin-SA conjugation, with as much as 10% or more for some aa-tRNAs. After biotinylation, the aminoacyl group of the aa-tRNA is stabilized and can react with SA at room temperature. It is with this temperature adjustment that we discovered that the biotin-SA conjugation can more quantitatively and robustly capture the aa-tRNA product than the traditional assay with a labeled amino acid (Table 1). 

While the label-free assay is not limited to the concentration of the amino acid substrate, it is also not limited to the concentration of the tRNA substrate. This is a clear advantage compared to the assay with a [3′-^32^P]-labeled tRNA, which is restricted to low concentrations of tRNA so as not to dilute out the radioactivity and de-sensitize signal detection. However, many human neurological disorders are associated with mutations in tRNAs, particularly those encoded by the mitochondrial genome. A notable example is the mutation in mt-tRNA^Leu^(UUR) associated with the MELAS (mitochondrial encephalomyopathy, lactic acidosis, and stroke-like episodes) disease [49], which decreases aminoacylation [50], possibly by decreasing the tRNA affinity to the cognate mitochondrial aaRS. To address this possibility, and to extend to other pathogenic mitochondrial tRNA mutations associated with mitochondrial diseases (e.g., in mitochondrial tRNA^Val^ [51]), the label-free assay would be preferable, because it can readily accommodate high concentrations of tRNA while maintaining the relative concentration of the aaRS low enough to detect aminoacylation. This flexibility with respect to the substrate concentration of both the amino acid and the tRNA substrate will provide a framework within which to apply the label-free assay to study mutant enzymes and tRNAs that are relevant to human health and disease and relevant to synthetic chemistry that aims at recoding the genetic code.

## Figures and Tables

**Table 1:** Quantification of aminoacylation of tRNA (%).

aa-tRNA	With Labeled Amino Acid	With Biotin-SA at 4 °C	With Biotin-SA at RT
Aminoacylation with the cognate aaRS enzyme			
Met-tRNA^Met/CAU^	32	41	26
Arg-tRNA^Arg/ICG^	27	43	28
Tyr-tRNA^Tyr/QUA^	33	33	27
Val-tRNA^Val/cmo5UAC^	23	40	35
Ala-tRNA^Ala/GGC^	35	59	49
Lys-tRNA^Lys/mnm5s2UUU^	16	30	27
Pro-tRNA^Pro/GGG^	24	30	23
Ser-tRNA^Ser/GCU^	–	51	32
Aminoacylation with dFx flexizyme			
*cis*-HxyPro-tRNA^Ser^	–	27	25
*trans*-HxyPro-tRNA^Ser^	–	10	10
Azetidine-tRNA^Ser^	–	24	20
Citrulline-tRNA^Ser^	–	50	38
NEM-Cys-tRNA^Ser^	–	29	25

Calculation of % of aminoacylation by the assay with a labeled amino acid is described in Materials and Methods, while calculation of % of aminoacylation by biotin-SA capture at 4 °C was based on data shown in Figure 2a,b and Figure 3b. As shown, biotinylation at RT underestimated the amount of aa-tRNA. Symbols and abbreviations: “–”, not determined; SA: streptavidin; and RT: room temperature.

## Supplementary Materials

The following are available online at https://www.mdpi.com/2073-4425/11/10/1173/s1, Figure S1: Aminoacylation kinetics of tRNA^Tyr^ monitored by the biotin-streptavidin conjugation assay. A concentration series of overexpressed tRNA^Tyr^ in a pool of total native tRNA was charged using a limiting amount of TyrRS. Aliquots of each reaction were quenched at 2, 4, 8, and 10 min and after ethanol precipitation 4.5 pmoles of the tRNA (containing ~1.5 pmoles of tRNA^Tyr^) was biotinylated, conjugated to streptavidin, and electrophoresed through a denaturing 12% PAGE/7M urea gel. Signals were calculated by multiplying the fraction of Tyr-tRNA^Tyr^ in each lane of the SYBR gold stained gel by the µM concentration of the input tRNA^Tyr^ in the aminoacylation reaction. These data were used to construct the plot in Figure 4.

## Abbreviations

aminoacyl-tRNA (aa-tRNA); streptavidin: SA; bovine serum albumin: BSA; dithiothreitol: DTT; ethylenediaminetetraacetic acid: EDTA; sodium acetate: NaOAc; 3,5-dinitrobenzyl ester: DBE; N-hydroxy-sulfo-succinimido-biotin: sulfo-NHS-biotin; polyacrylamide gel electrophoresis: PAGE; N-ethylmaleimide: NEM.
