# Supplementary material for: A Label-Free Assay for Aminoacylation of tRNA
Source: Genes (Basel). 2020 Oct 7;11(10):1173. doi: 10.3390/genes11101173 (PMC7601589; doi:10.3390/genes11101173)
Supplement: Supplementary file 1 [file genes-11-01173-s001.pdf]

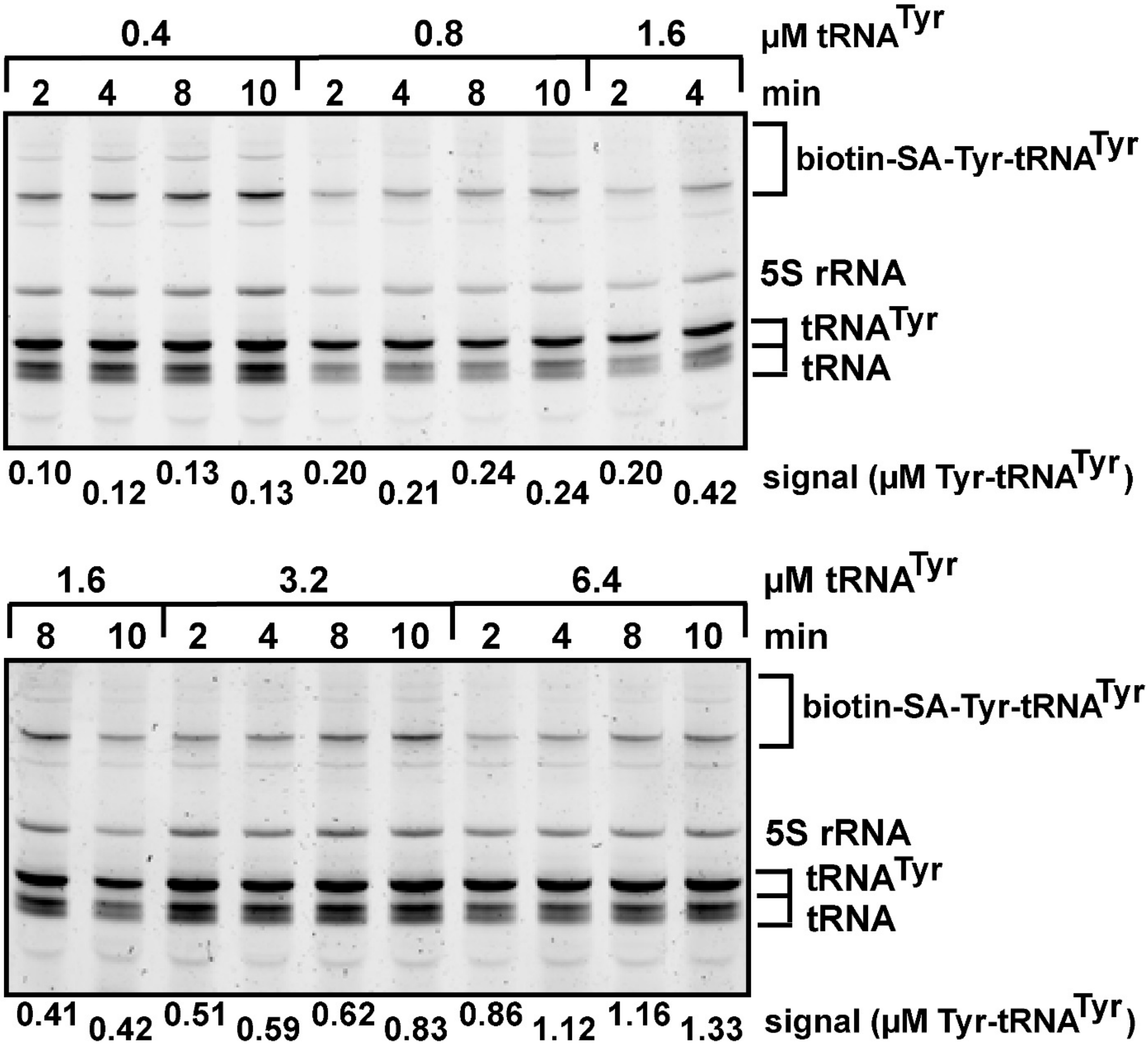

Figure S1. Aminoacylation kinetics of $tRNA^{Tyr}$ monitored by the biotin-streptavidin conjugation assay. A concentration series of overexpressed $tRNA^{Tyr}$ in a pool of total native tRNA was charged using a limiting amount of TyrRS. Aliquots of each reaction were quenched at 2, 4, 8, and 10 min and after ethanol precipitation 4.5 pmoles of the tRNA (containing ~1.5 pmoles of $tRNA^{Tyr}$) was biotinylated, conjugated to streptavidin, and electrophoresed through a denaturing 12% PAGE/7M urea gel. Signals were calculated by multiplying the fraction of Tyr-$tRNA^{Tyr}$ in each lane of the SYBR gold stained gel by the µM concentration of the input $tRNA^{Tyr}$ in the aminoacylation reaction. These data were used to construct the plot in Figure 4.
